# Supplementary material for: RNA Editing Enzyme ADAR1 Regulates METTL3 in an Editing Dependent Manner to Promote Breast Cancer Progression via METTL3/ARHGAP5/YTHDF1 Axis
Source: Int J Mol Sci. 2022 Aug 25;23(17):9656. doi: 10.3390/ijms23179656 (PMC9456332; doi:10.3390/ijms23179656)
Supplement: Supplementary file 1 [file ijms-23-09656-s001.zip › ijms-1868218-supplementary.pdf]

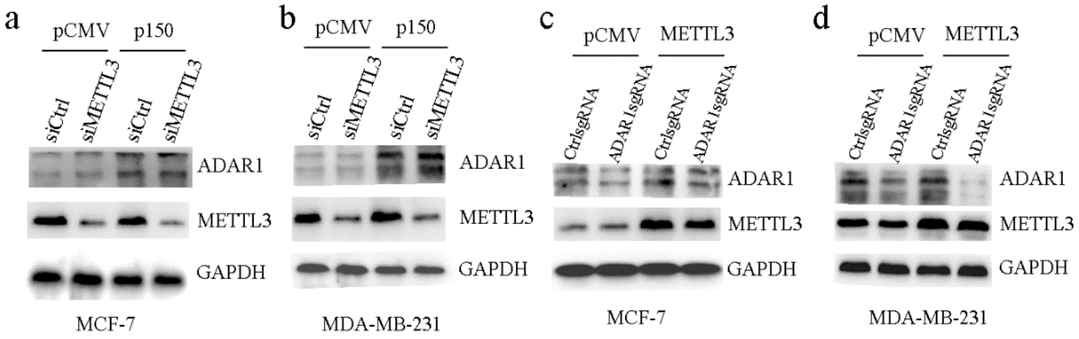

Figure S1. Protein expression levels of ADAR1 and METTL3 in breast cancer cells transfected with or without siRNAs and plasmids described in Figure 3.

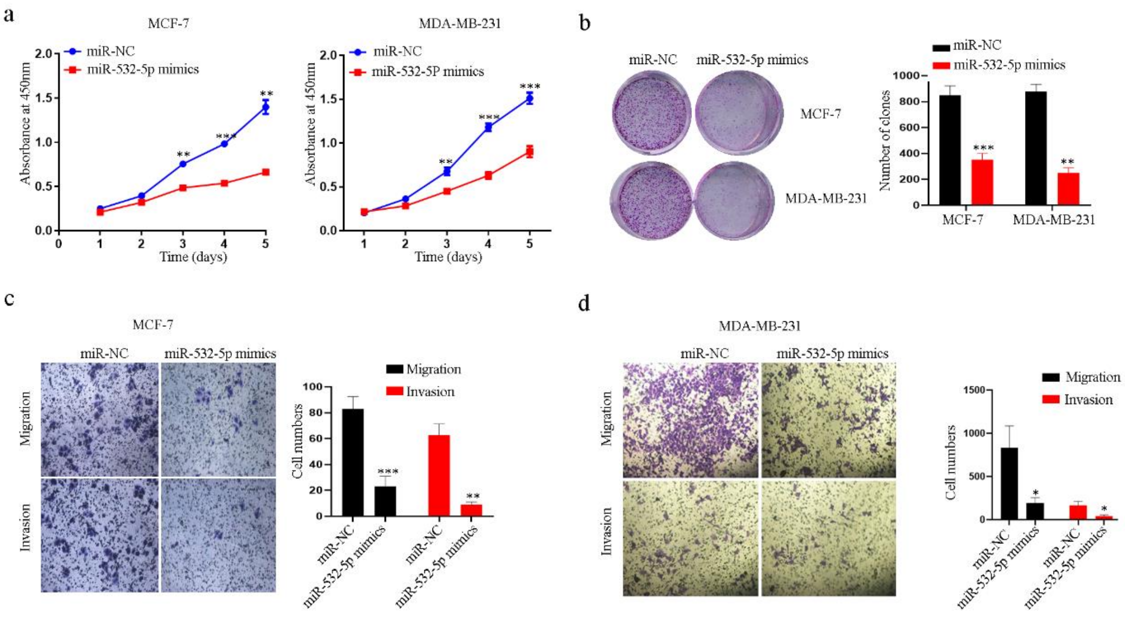

Figure S2. miR-532-5p inhibits cell proliferation, colony formation, cell migration and invasion of breast cancer cell lines.

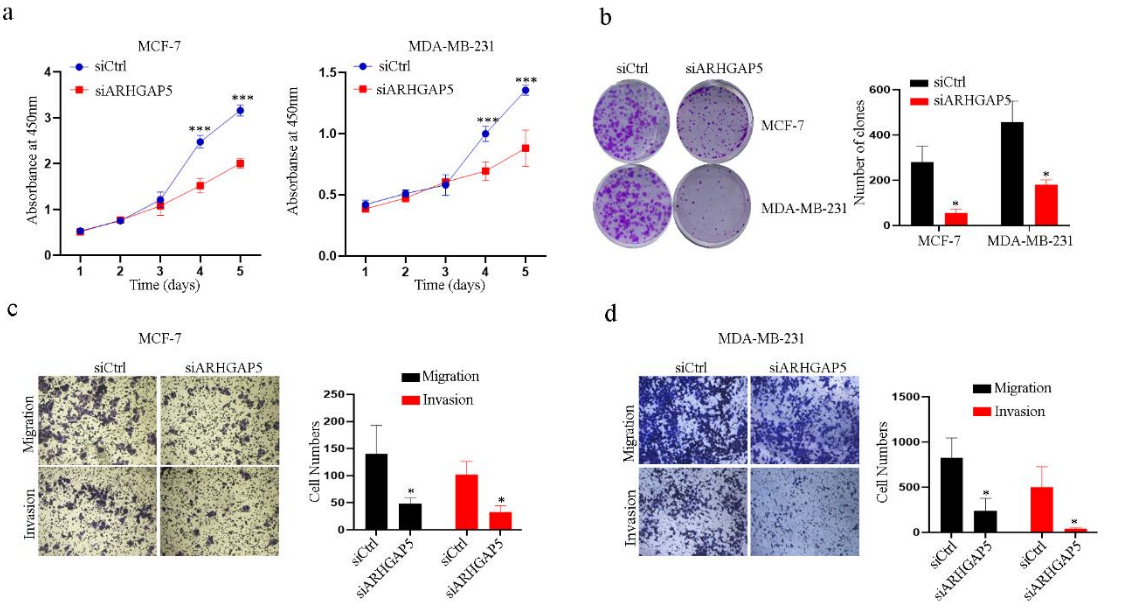

Figure S3. Knockdown of ARHGAP5 inhibits cell proliferation, colony formation, cell migration and invasion of breast cancer cell lines.

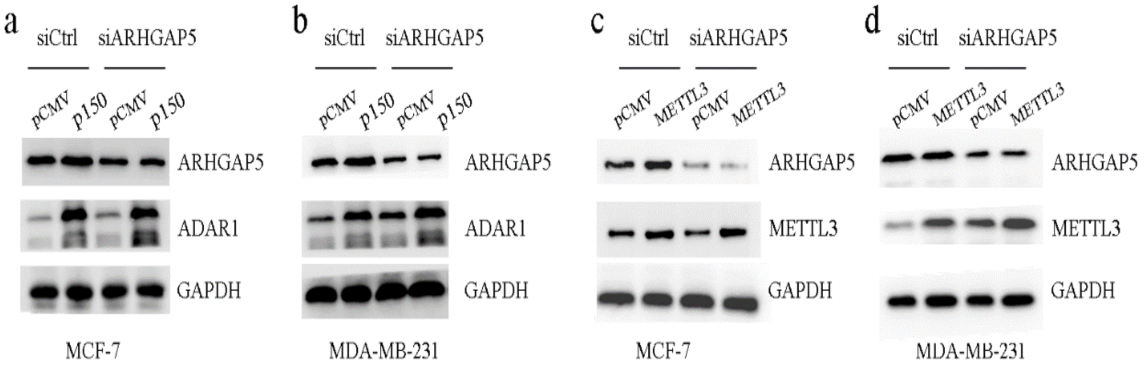

Figure S4. Protein expression levels of ARHGAP5, ADAR1 and METTL3 in breast cancer cells transfected with or without siRNAs and plasmids described in Figure 6.

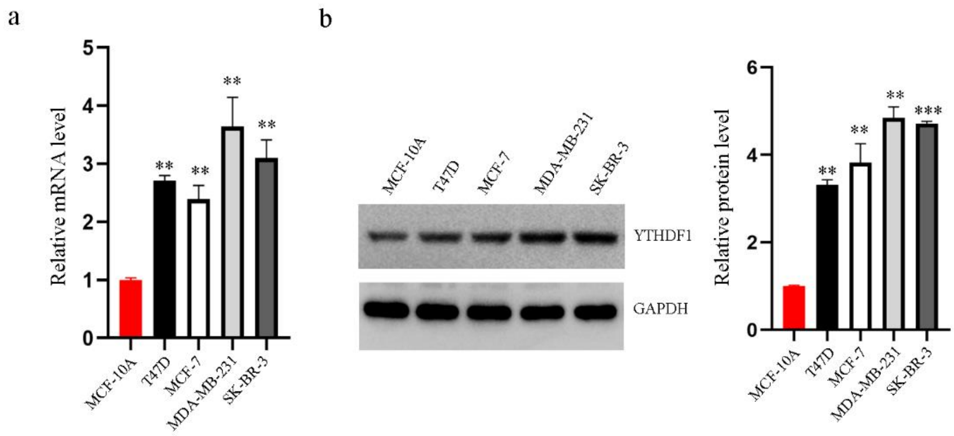

Figure S5. YTHDF1 is highly expressed in breast cancer cell lines.

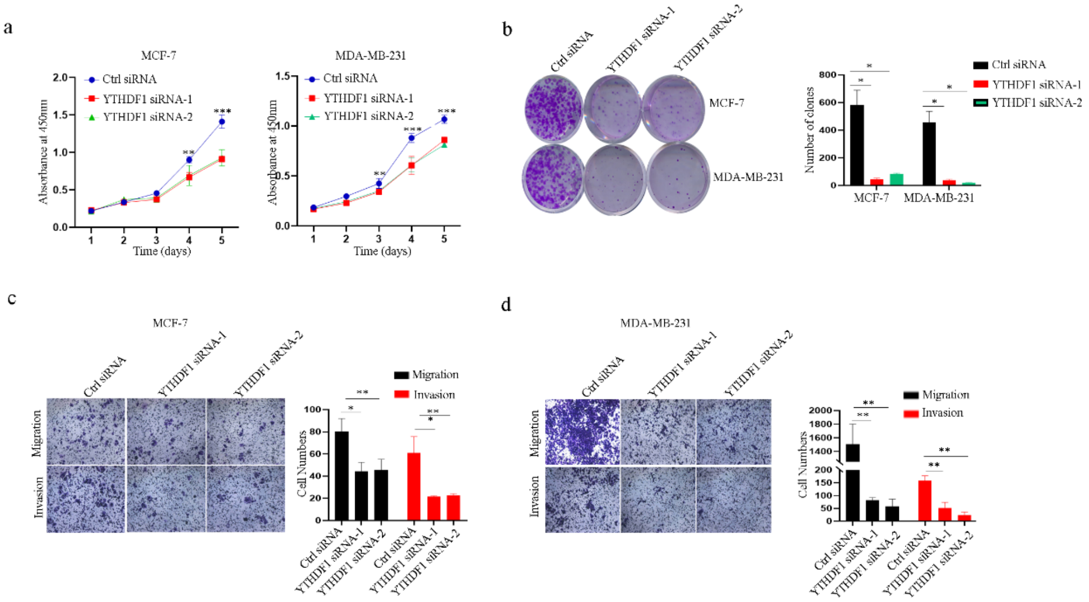

Figure S6. Knockdown of YTHDF1 inhibits cell proliferation, colony formation, cell migration and invasion of breast cancer cell lines.

Table S1 Oligo sequence used in the experiments

Oligo name	Oligo sequence (5′→3′)
**siRNA target oligo**	
siYTHDF1-1	GATACAGTTCATGACAATGA
siYTHDF1-2	GAAACGTCCAGCCTAATTCT
siYTHDF2	GAACGTCAAGGTCGTGGGAAA
siYTHDF3	TACGGCATATTCGCTTAGAAA
siARHGAP5	AGAUCAUAAUAUCAAUCUATT
siMETTL3	CUGCACUUCAGACGAAUUATT
**PCR primers**	
YTHDF1-F	ATGTCGGCCACCAGCGTGGACA
YTHDF1-R	TCATTGTTTGTTTCGACTCTGC
GAPDH-F	TCTTAAGAAGACGACGGCTTCAG
GAPDH-R	TTGCTCTCTCACTTGTCCTCGAT
ARHGAP5-F	AGGGAAGCTCAACGTAGATGG
ARHGAP5-R	ATGATCCACGCATTCATCACAT
ADAR1-F	CATCAGCGGGCTGTTAGAAT
ADAR1-R	CTTGGCCACTTTCTTGCTTC
METTL3-F	AGCCTTCTGAACCAACAGTCC
METTL3-R	CCGACCTCGAGAGCGAAAT
